# Descriptive Epidemiology of Fall-Related Injuries Among Older Adults in Ontario, Canada

**DOI:** 10.1093/geroni/igab046.2897

**Published:** 2021-12-17

**Authors:** Nicolette Lappan, Aleksandra Zecevic, Yu Ming, Susan Hunter, Andrew Johnson

**Affiliations:** 1 University of Western Ontario, University of Western Ontario, Ontario, Canada; 2 Western University, London, Ontario, Canada

## Abstract

The number of older adults is growing rapidly in the province of Ontario meaning there will be more fall-related injuries (FRIs) in coming decades. Falls are the leading cause of injury-related hospitalizations in Canada. The purpose of this study was to describe the prevalence, circumstances, types, and locations of FRIs among older adults in Ontario. Using a population-based retrospective design, we analyzed secondary data from three health administrative databases (NACRS, DAD, RPDB) for 2010-2014. Older adults (≥ 65 years) admitted to an emergency department (ED) with a combined diagnosis of ICD-10-CA codes for a fall (W00-W19) and injury (S00-S99 or T00-T14) were selected. Descriptive statistics were performed in R and rates were reported per 100,000 population. There were 304,610 FRI ED admissions (3,089/100,000) and 143,210 patients (47.0%) were subsequently hospitalized (1,452/100,000). Females accounted for 63.0% ED and 61.2% hospital admissions. Age-specific rates increased with age at both ED (2,208/100,000 in 65-69 group, 6,552/100,000 in 90+ years old) and hospital (698/100,000 in 65-69 group, 4,364/100,000 in 90+ years old). Females had higher rates of ED (3,503 vs. 2,572/100,000) and hospital (1,598 vs. 1,270/100,000) admissions than males. The most common injury types at the ED were fractures (1,234/100,000), superficial injuries (719/100,000), other or unspecified injuries (572/100,000), open wounds (498/100,000), and sprains, strains, and tears (162/100,000). FRIs are a considerable problem for older adults and better injury prevention strategies are needed for all female age groups, the 90+ year age group of both genders, and fractures.

